# Effects of L-dopa during Auditory Instrumental Learning in Humans

**DOI:** 10.1371/journal.pone.0052504

**Published:** 2012-12-21

**Authors:** Tina Weis, Sebastian Puschmann, Andre Brechmann, Christiane M. Thiel

**Affiliations:** 1 Biological Psychology, Department of Psychology, Carl-von-Ossietzky University Oldenburg, Oldenburg, Germany; 2 Special-Lab Non-invasive Brain Imaging, Leibniz Institute for Neurobiology, Magdeburg, Germany; 3 Research Center Neurosensory Science, Carl-von-Ossietzky University Oldenburg, Oldenburg, Germany; University of Cambridge, United Kingdom

## Abstract

The dopaminergic neurotransmitter system is critically involved in promoting plasticity in auditory cortex. We combined functional magnetic resonance imaging (fMRI) and a pharmacological manipulation to investigate dopaminergic modulation of neural activity in auditory cortex during instrumental learning. Volunteers either received 100 mg L-dopa (Madopar) or placebo in an appetitive, differential instrumental conditioning paradigm, which involved learning that a specific category of frequency modulated tones predicts a monetary reward when fast responses were made in a subsequent reaction time task. The other category of frequency modulated tones was not related to a reward. Our behavioral data provides evidence that dopaminergic stimulation differentially impacts on the speed of instrumental responding in rewarded and unrewarded trials. L-dopa increased neural BOLD activity in left auditory cortex to tones in rewarded and unrewarded trials. This increase was related to plasma L-dopa levels and learning rate. Our data thus provides evidence for dopaminergic modulation of neural activity in auditory cortex, which occurs for both auditory stimuli related to a later reward and those not related to a reward.

## Introduction

The cortical representation of sensory stimuli is modulated by experience [1]–[5]. This change in primary and secondary sensory cortices is known as cortical plasticity, which is a prerequisite for lifelong learning and recovery after damage. Auditory cortex plasticity has mostly been investigated using aversive conditioning paradigms, where a previously neutral auditory stimulus (conditioned stimulus, CS) acquires significance through its prediction of a future aversive event such as an electric foot shock (unconditioned stimulus, US). Several studies provide evidence for learning related changes after only a few pairings of the auditory stimulus with the foot shock indicated by receptive-field shifts [6]–[9]. Studies using instrumental appetitive conditioning tasks, in which animals learn to execute an appropriate response to a specific auditory stimulus (CS+) in order to gain a reward, whereas responses to other auditory stimuli (CS−) are not rewarded, show similar learning related effects [10]–[16].

The neural mechanisms of reinforcement learning explained by theoretical models (e.g. [17]) point to a crucial role of dopaminergic neurons within the ventral tegmental area (VTA) for the development of learning related plasticity. This assumption is further supported by several studies reporting significant effects of VTA activity on learning and long-term potentiation [18], [19]. In line with these studies, it has been shown that the dopaminergic neurotransmitter system is involved in enhancing plasticity in auditory cortex [13], [20]–[22]. Studies in rodents indicate that the auditory cortex receives significant inputs from the VTA [23]. Furthermore, Bao and colleagues [21] showed that the simultaneous presentation of a pure tone with an electric stimulation of the VTA increased the spatial representation of this specific tone in auditory cortex. Blocking dopaminergic D1 and D2 receptors inhibited this effect. Thus, this study demonstrated that ventral tegmental dopamine-mediated activity enables the reorganization of auditory cortex. In vivo microdialysis provides further evidence for an increase of homovanillic acid, a major dopamine metabolite, in auditory cortex during the first day of aversive associative auditory learning, but not in later re-learning sessions on the following days [20]. In other words, dopamine is released in early stages of learning and dopaminergic D1/D5 receptors may be critical. Administration of the D1/D5 dopamine receptor agonist SKF-38393 before or shortly after initial training of a foot shock avoidance task did not impact on initial acquisition performance but increased frequency modulated (FM) tone discrimination performance during retraining on following days. Thus, increased dopaminergic activity at the time point of initial learning promotes later memory formation via gene activation and synaptic remodeling [24].

In humans, learning-dependent modulations of auditory cortex responses have been reported previously in classical conditioning paradigms [25]–[27]. Similar results were found in an instrumental conditioning paradigm, where increased neural activity in auditory cortex, VTA and nucleus accumbens was found when subjects learned that a specific class of auditory stimuli predicted a reward for fast responses in a reaction time task [28]. Thus, representations of auditory stimuli differed depending on learning reward associations, and neural activity from dopaminergic midbrain regions may contribute to these plastic changes. A recent study by Guitart-Masip et al. [29] in human volunteers provides evidence that dopaminergic modulation of neural activity is mainly seen in conditions that combine the anticipation of a reward with an instrumental requirement. Levodopa (L-dopa) only increased neural activity in dopaminergic brain regions in conditions, where a response had to be made to obtain a reward as compared to those that required refraining from responding to obtain a reward [29].

The role of the dopaminergic system has primarily been investigated in classical conditioning paradigms (e.g. [30], [31]). However, some recent studies point to an involvement of dopamine in instrumental learning and have shown that boosting dopamine increases the likelihood of choosing the most rewarding stimulus [32]–[34]. Further, a recent study by Wunderlich et al. [34] investigated, how two different models of reinforcement learning contribute to choice behavior and showed that dopamine enhances model-based choices over the model-free alternative.

To provide direct evidence that dopaminergic neurotransmission critically modulates representations of relevant stimuli in human auditory cortex, we performed a pharmacological functional magnetic resonance imaging (fMRI) experiment and used an instrumental learning paradigm. Volunteers either received L-dopa or placebo in an appetitive instrumental conditioning paradigm, which involved learning that a specific class of FM tones defined by a certain target feature predicts a monetary reward, when a fast response was made in a succeeding reaction time task [28]. In other words, a reward was obtained for those trials, in which the conditioned stimulus contained the target feature and in which the subsequent reaction with button press was fast and correct. The task thus enables the separation of learning about target features of sensory stimuli and the subsequent operant behavior to obtain a reward.

We focused our analysis on the phase of the learning trial, where FM tones were presented and subjects learnt to categorize auditory stimuli as associated with a reward or not associated with a reward. We hypothesized that L-dopa would increase this neural activity in the auditory cortex and in dopaminergic midbrain areas (such as substantia nigra/VTA) compared to placebo. Furthermore, we hypothesized that L-dopa would increase the efficiency of the participants in solving the instrumental conditioning task by improving categorization of auditory stimuli (i.e. learning rate) as well as the speed of operant conditioning indicated by reaction times (RTs).

## Materials and Methods

### Subjects

This study was conducted in accordance with the Declaration of Helsinki [35] and the experiments were approved by the ethics committee of the University of Magdeburg. Written informed consent was obtained from 66 normal hearing, healthy volunteers. All participants were right-handed as indexed by a handedness inventory [36], had no history of neurological or psychiatric disease and were not on any kind of medication (except for contraceptives). A clinical evaluation was first carried out to ensure that the subjects had no conditions contraindicative for L-dopa administration. Participants were asked to avoid excessive alcoholic intake on the evening before the test session and to refrain from eating, smoking and drinking coffee for two hours prior to the experiment. Four subjects were excluded from all further analyses due to non-compliance with task instructions, five subjects due to excessive head movement during fMRI scanning (overall head movement >3.5 mm, scan-to-scan movement >2 mm). Two more subjects were excluded after analysis of blood samples due to non-detectable L-dopa levels. The group size remaining in the analysis was 55 subjects in total, n = 28 for placebo (20 male, 8 female, mean age = 28±1 years), and n = 27 for L-dopa (18 male, 9 female, mean age = 29±1 years). All participants were at least 25 years old (exclusion criteria patient information sheet).

### Pharmacological Manipulation

We used a double-blind placebo-controlled drug administration technique in a between-subject design. Participants were randomly allocated to one of two groups either given placebo (solution containing glucose dissolved in water) or Madopar LT® (100 mg L-dopa/25 mg benserazide, dissolved in water), orally 30 minutes before starting the fMRI measurement. Two prior studies indicate that different dosages of L-dopa result in a dose-dependent non-linear plasticity effect in human motor cortex [37], [38]. Since in those studies medium dosage (100 mg) prolonged facilitatory and inhibitory plasticity, whereas low and high dosage (25 mg and 200 mg, respectively) abolished plasticity effects, we used in our study a dose of 100 mg. FMRI scanning started approximately 30 minutes after drug intake, since it is known that L-dopa reaches peak plasma concentrations after about half an hour [39]. Many studies using L-dopa/benzerazide 100 mg started their measurements one hour after drug intake [32], [37], [38]. However, in contrast to these studies, we used the water-dispersible Madopar LT®, which reaches peak plasma concentration faster than the conventional Madopar tablet, i.e. after around 30 minutes [40]–[42]. Blood samples of each participant were taken before entering the scanner.

### Effects of L-dopa on Blood Flow

Since fMRI measures the endogenous BOLD response, it relies on hemodynamic coupling between neural activity and regional changes in blood flow and oxygenation. Therefore, it is important to take into account possible drug-induced changes in neural-hemodynamic coupling. A study of Rao et al. [43] shows that methylphenidate, which enhances extracellular dopamine levels comparable to L-dopa, did not alter the local neural-hemodynamic coupling. Furthermore, a study using L-dopa in a rat model of Parkinson’s disease [44] shows that sham lesioned animals, receiving a single dose of L-dopa showed no changes in regional cerebral blood flow. However, some studies in humans have reported changes in blood flow after L-dopa administration. Montastruc et al. [45] performed a SPECT study in Parkinson’s patients as well as normal subjects treated with 250 mg L-dopa and showed a significant increase in blood flow in both groups. Furthermore, Leenders et al. [46] used an increased dose of drug over ten days up to a maximum daily dose of 500 mg in normal control subjects and Parkinson’s patients and also found an increase in blood flow. Note, however, that those studies used a significantly higher dose of L-dopa. Further, there is evidence from cocaine that changes in cerebral blood flow were not associated with a modulation of the BOLD signal [47]. We therefore assume that changes in BOLD activity under L-dopa are not due to changes in neural hemodynamic coupling or cerebral blood flow.

### Task

We used an appetitive instrumental conditioning paradigm, where participants had to learn the association of a specific category of FM tones with the chance to gain a monetary reward in a subsequent reaction time task. The task is similar to the monetary incentive delay task introduced by [48]–[50]. The task was employed in several fMRI studies an yielded strong increases in neural activity in dopaminergic brain regions during reward anticipation [18], [51], [52]. The delay enables separation of neural responses to reward predicting stimuli and the later reward phase. Note that in contrast to previous studies, our paradigm involved sensory learning, since conditioned stimuli had to be categorized into CS+ and CS− trials. The focus in this study is only the neural activity during reward anticipation; analysis of neural activity during the reward delivery phase will be published elsewhere.

**Figure 1:**
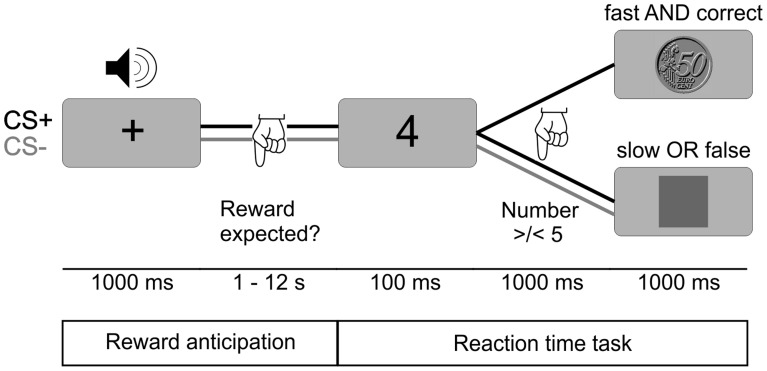
Paradigm. In the appetitive instrumental conditioning paradigm each trial started with an FM tone which differed in various categories. In half of the trials participants had the chance to gain a monetary reward (CS+), whereas the other half of the trials remained unrewarded (CS−). The main task for the participants was to find out by trial and error which feature of the FM tones predicts a reward. After each tone, they had to state their current reward expectancy for the upcoming trial via key press. To receive this reward, participants had to solve a simple reaction time task, in which they had to indicate whether a number shown on a screen was smaller or larger than five. If their answer in the number comparison task was fast and correct, they were rewarded with 50 Euro-Cent, if it was a CS+ trial. Slow and incorrect answers in CS+ trials resulted in no reward (indicated by a grey square). In the other half of the trials (CS−), participants were never rewarded, independent of the correctness and speed of their response.

At the beginning of each trial, an FM tone was presented, which indicated whether the upcoming trial was potentially rewarded with 50 Euro-Cent (CS+ trials) or not (CS− trials) (see Figure 1). The FM tones differed in five stimulus dimensions each with two different levels: direction, duration, loudness, frequency range, and modulation rate (for further details on stimuli see below). Participants were informed that one category of these FM tones predicts a reward, but had to find out the relevant feature by trial and error. The relevant reward-prediction feature was duration (400 ms vs. 800 ms). Whether the short or long duration FM tones predicted the reward was randomized across subjects. The number of short and long duration FM tones (i.e. CS+ and CS− trials) was the same. To assess the individual learning rate, participants had to indicate via button press after each tone if they expected a reward in the upcoming trial or not. They had to press the left button with the index finger if they expected a reward and the right button with the middle finger if they did not expect a reward.

Rewards were obtained for fast responses in a subsequent reaction time task. Here, participants had to indicate by button press if the number (1, 4, 6 or 9) presented on the screen for 100 ms was smaller or larger than five (index finger for ‘smaller’, middle finger for ‘larger’). Based on an individual reaction time threshold, which was determined before the experiment (see below), fast and correct responses in CS+ trials were financially rewarded [18], [53]. The reward was indicated by a 50-Euro-Cent coin displayed on the screen at the end of each trial 1.5 seconds after the onset of the number presentation. In CS− trials no reward was given independent of the subject’s answer, and was signaled by a neutral feedback (grey square). The same feedback was also given for slow or incorrect answers in CS+ trials. A temporal jitter was used between the FM tone and the reaction time task in steps of 1.5 seconds ranging from 4.5 to 12 seconds. The inter-trial-interval ranged from 3.0 to 12.0 seconds also in steps of 1.5 seconds. A fixation cross was presented in the middle of the screen during all delays and during presentation of the FM tones. The total experiment comprised 160 trials in 42 minutes. Participants received payment of the amount of gained reward at the end of the experiment.

To obtain individual reaction time thresholds for fast responses, participants performed the reaction time task prior to entering the MRI scanner. As during scanning, they had to indicate via button press whether the presented number was smaller or larger than five. The 80% value of reaction time in 80 trials was calculated and taken as a starting threshold for gaining a potential reward in the following paradigm during fMRI measurement. This reaction time threshold was 560±94 ms for L-dopa and 559±64 ms for placebo (*p = 0.9*). During the task in the scanner, the reaction time threshold was individually adjusted relatively to the performance of the subjects to guarantee that subjects received rewards even in case of declining or increasing RTs over time. Adjustments consisted of increasing and decreasing the reaction time threshold by 50 ms in case of 10 unrewarded and rewarded trials in succession, respectively. All experimental control software was programmed in MATLAB (The MathWorks, Inc., Natick, MA, USA) using Cogent 2000.

### Stimuli

Each stimulus dimension (frequency range, modulation rate, loudness, direction and duration) consisted of two principle levels. There was a low and a high frequency band, each containing five onset frequencies separated by half-tone steps (500 Hz, 530 Hz, 561 Hz, 595 Hz, 630 Hz/1630 Hz, 1732 Hz, 1826 Hz, 1915 Hz, 2000 Hz). Frequencies varied either with 0.25 octaves/second or 0.5 octaves/second. Overall sound level was individually adjusted under scanner noise for both louder and quieter sounds differing approximately by 10 phon. Sound duration was either 400 ms (short) or 800 ms (long). The modulation direction was either rising or falling. In total, the combination of all possible values of the five dimensions resulted in 160 different stimuli; with eighty of them predicting a potential reward (all short or all long FM tones).

### fMRI Data Acquisition

FMRI data acquisition was performed on a 3 T Siemens MAGNETOM Trio MRI scanner (Siemens AG, Erlangen, Germany) with an eight-channel head array. Key-presses were recorded using a MR-compatible response keypad (LUMITouch, Photon Control Inc., Burnaby, BC, CDN). Acoustic stimuli were delivered by MR compatible headphones (MR confon OPTIME 1, MR confon GmbH, Magdeburg, Germany). During functional measurements 1680 T_2_*-weighted gradient echo planar imaging (EPI) volumes (time of repetition (TR) = 1.5 ms, time of echo (TE) = 30 ms, flip angle 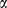 = 80°, field of view (FoV) = 192×192 mm^2^, voxel-size = 3.0×3.0×3.0 mm^3^) were obtained within one session. Volumes consisted of 24 interleaved slices (gap of 0.3 mm) ranging from the anterior cingulate cortex dorsally to the inferior colliculus in the brain stem. After the experimental task a high-resolution T_1_-weighted structural volume was obtained from each subject.

### Behavioral Data Analysis

Three behavioral measures were of interest: i) learning rates, i.e. correct expectation of reward, ii) speed of instrumental responding in the reaction time task and iii) RTs after tone presentation. The expectation of reward was indicated by the subjects after presentation of the FM tone and reflects whether the tone-reward association was learnt. For each subject, we determined the individual learning curve using the cumulative sum of correct responses as function of experiment duration according to Gallistel et al. [54]. A linear regression was performed on each cumulative learning curve to specify the individual degree of learning, indexed by the slope of the regression line. Slopes were averaged and compared across groups by means of an unpaired t-test. To investigate whether learning impacts on neural activity, the slope of the cumulative learning curve for each subject was entered into the fMRI data analysis (see below).

The RTs in the number comparison task were the second behavioral measure of interest. Previously, it was shown that RTs in the number comparison task following a CS+ stimulus were significantly shorter than those following a CS− stimulus after learning the tone reward association [18], [28]. To analyze RTs over time as a function of *condition* (CS+/CS−) and *group* (L-dopa/placebo), we extracted for each subject the slope of RTs changes to CS+ and CS− trials and analyzed these with an ANOVA with the within subject factor *condition* and the between subject factor *group*.

We performed an additional analysis of RTs after tone presentation using an ANOVA with the within subject factor *condition* (CS+/CS−) and the between subject factor *group* (L-dopa/Placebo). In contrast to the ANOVA in the number comparison task, in which the slopes of the RT changes were used, we here used the median RTs of each subject.

### Physiological and Subjective Data Analysis

We measured pulse rate, blood pressure and subjective drug effects in both groups before and 30 minutes after drug administration. Subjective drug effects were assessed with visual analogue scales for the three factors ‘alertness’, ‘contentedness’, ‘calmness’ [55]. Moreover, the subjects completed a symptom checklist asking for known negative side effects of L-dopa. Mean subjective rating scores pre- and post- (i.e. prior to scanning) drug as well as pulse rate and blood pressure were analyzed for a *group by time* interaction with ANOVAs for repeated measures, followed by post hoc t-tests where appropriate.

### fMRI Data Analysis

MRI data were processed and analyzed using SPM8 (FIL, Wellcome Trust Centre for Neuroimaging, UCL, London, UK). To correct head motion, the functional time series were spatially realigned and unwarped. The structural T_1_-weighted volume was registered to a mean functional image and segmented in order to obtain spatial normalization parameters. Using these parameters, functional and structural images were normalized to the Montreal Neurological Institute (MNI) template brain. Finally, normalized functional volumes were smoothed with a three-dimensional Gaussian kernel of 4 mm full-width-half-maximum.

The single subject model contained nine regressors: two regressors for BOLD responses to CS+ and CS− tones with correct reward expectations, two further regressors for CS+ and CS− tones with incorrect reward expectations and an additional regressor for missed responses. The last four regressors modeled the feedback phase. For CS+ trials one regressor accounted for receipt of reward after fast responses, another one for neutral feedback after slow responses. For CS− trials we used one regressor modeling neutral feedback in CS− trials after correct responses. Neutral feedback after response errors, i.e. wrong or no button press in the reaction time task, was pooled in one additional regressor. Time series in each voxel were high-pass filtered to 1/128 Hz and modeled for temporal autocorrelation across scans with an AR(1) process.

Statistical data analysis was focused on neural responses to FM tones during the reward anticipation phase. Single subject contrasts coding for FM tones in CS+ and CS− trials with correct reward expectations were entered into a full factorial ANOVA design for further analysis. Only correct reward expectations entered into the ANOVA, since the regressor for wrong reward expectations contained less than ten trials in at least thirty subjects. The following factors were included in the ANOVA model: *group* (L-dopa/placebo) and *condition* (CS+/CS−). Within this full factorial ANOVA model we calculated the t-contrast *L-dopa > placebo*, *CS+ > CS*− and the *group×condition* interaction.

Two additional linear regression analyses were performed to relate BOLD activity to i) learning rates and ii) L-dopa levels. To investigate the relationship between learning curves and BOLD activity under placebo and L-dopa, we included the slope of the cumulative learning curves for each individual subject as covariate in a two sample t-test. We used a single subject contrast coding for FM tones in CS+ as well as CS− trials for correct reward expectations (i.e. these two regressors were contrasted against an implicit baseline). To investigate the relationship between L-dopa levels and BOLD activity, we included the L-dopa levels from each individual blood sample of subjects in the L-dopa group as covariate in a one sample t-test with the same single subject contrast as for the linear regression related to learning rates.

Results of all analyses were thresholded at a single voxel value of *p<0.001* and are reported corrected for the whole brain or for regions of interest at *p<0.05*, established with a Monte Carlo voxel-cluster threshold technique (see program AlphaSim by Douglas Ward in AFNI software (http://afni.nimh.nih.gov/pub/dist/doc/manual/AlphaSim.pdf; [56]). All tables state peak MNI coordinates, cluster volume in voxel (2×2×2 mm), Z-values, and corresponding brain regions. All clusters were identified using a corrected alpha level of 0.05 (voxelwise *p<0.001*; cluster-size > = 64 voxels, for total scanning volume; cluster-size > = 18 voxels, for small volume correction, indicated by asterisks). To further visualize significant effects in regions of interest as a function of group and condition, we extracted average beta values in a sphere with a radius of 6 mm around the activation peak maxima.

Regions of interest included the left and right auditory cortex, the left and right nucleus accumbens and the substantia nigra/VTA, which were previously shown to be strongly related to learning the reward-predicting auditory stimulus class [28]. These regions were combined into one mask, which was used for correction of multiple comparisons in regions of interest. The left and right nucleus accumbens were defined as a sphere of radius 6 mm around [x, y, z] = [9, 6, −9] and [x, y, z] = [−6, 0, 6] in MNI space according to Daniel and Pollmann [57]. The substantia nigra/midbrain region was defined by a sphere of radius 6 mm around [x, y, z] = [6, −21, −12] according to Wittmann et al. [18]. The left and right auditory cortex mask was derived from data of Puschmann et al. [28] by using the contrast *sound > baseline* masked with the region of superior temporal gyrus and Heschl’s gyrus (as included in the WFU PickAtlas extension for SPM [58], *p<0.001* (uncorrected)).

## Results

### Physiological and Subjective Measurements

Plasma L-dopa levels were 0.93±0.13 mg/l 30 minutes after drug intake, endogenous L-dopa levels in the placebo group were all below the detection limit. The results are comparable with a prior study on the effect of a single dose of 100 mg L-dopa on blood dopamine levels in healthy participants by Dingemanse and colleagues [39]. There were no significant differences between the two groups with respect to subjective ratings of alertness, contentness and calmness in the Bond and Lader Rating Scale (*p>0.1*). None of the subjects reported any side effects of L-dopa. There was also no significant change in pulse rate (*p>0.5*) neither in the L-dopa nor in the placebo treated group. However, regarding the blood pressure, there was a significant interaction in the diastolic (F(1,52) = 5.16, *p>0.02*) and a tendency for interaction in the systolic pressure (F(1,52) = 2.2, *p = 0.1*) with an increase in blood pressure in the placebo group and a slight decrease for L-dopa (see SI Table S1).

### Behavioral Data

**Figure 2:**
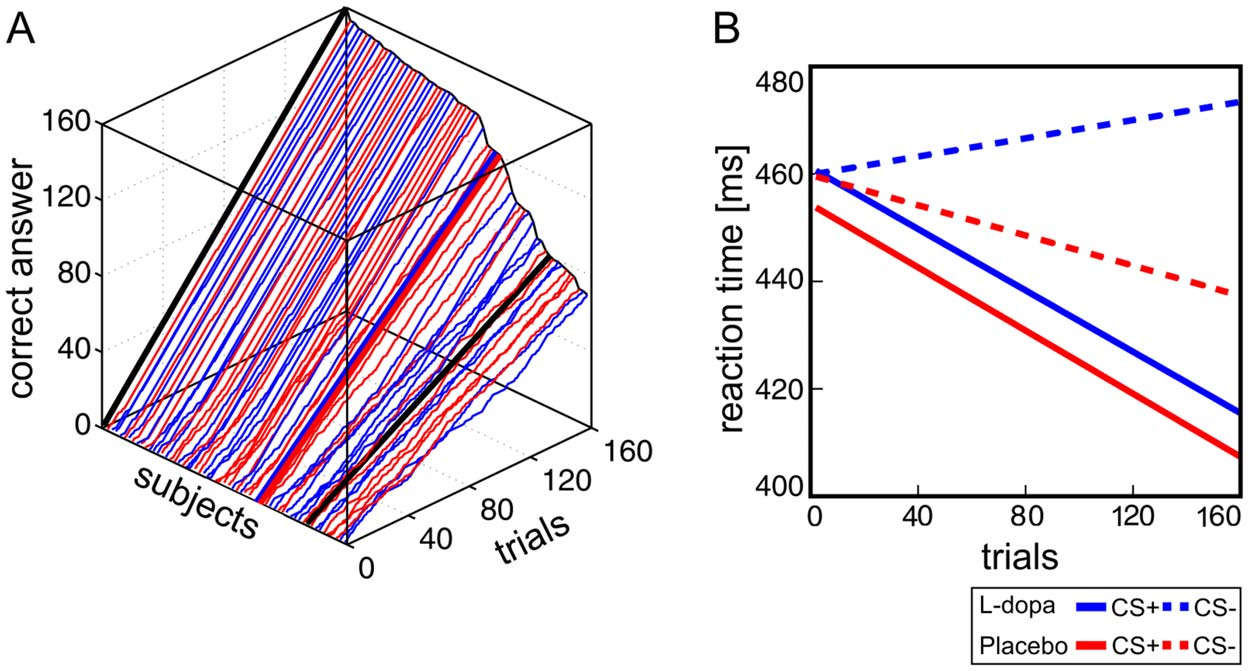
Behavioral data. (A) Learning curves: Cumulative sum of correct answers to reward expectations as function of trial number for each individual subject. Bold lines (blue and red) indicate the mean of each group (L-dopa and placebo, respectively). Bold black lines indicate 100% and 50% (chance) correct answers. (B) Speed of instrumental responding: Reaction times in the number comparison task in rewarded (CS+, solid lines) and unrewarded (CS−, dashed lines) as function of trial number for each group.

Learning rates. Figure 2A illustrates the individual learning rates, i.e. the cumulative sums of correct reward expectations indicated by the subject after presentation of the FM tones. Learning rates were similar for placebo and L-dopa treated subjects (T(1,53) = 0.14, *p = 0.8*).

Reaction times. An additional analysis of RTs after tone presentation shows no significant differences for the main effect of *group* (L-dopa/placebo) (F(1,26) = 0.43, *p = 0.51*) or *condition* (CS+/CS−) (F(1,26) = 1.34, *p = 0.31*) nor an interaction of *group x condition* (F(1,26) = 1.06, *p = 0.31*).

Speed of instrumental responding. Figure 2B displays RTs in the number comparison task for CS+ and CS− trials as a function of trial number in both groups. There was a decrease in RTs over time to CS+ trials in both groups (main effect of condition F(1,53) = 20.27, *p<0.001*) and a tendency for a significant group by condition interaction (F(1,53) = 3.53, *p = 0.065*). This was driven by the L-dopa group, where RTs in the number comparison task even increased over time in CS− trials, i.e. in trials, where no reward could be obtained for fast responding. Note that the amount of reward received in both groups was similar (L-dopa: 31±0.87 Euro, placebo: 31.87±0.56 Euro, T(1,53) = −0.84, *p = 0.4*).

### Functional MRI Data

**Figure 3:**
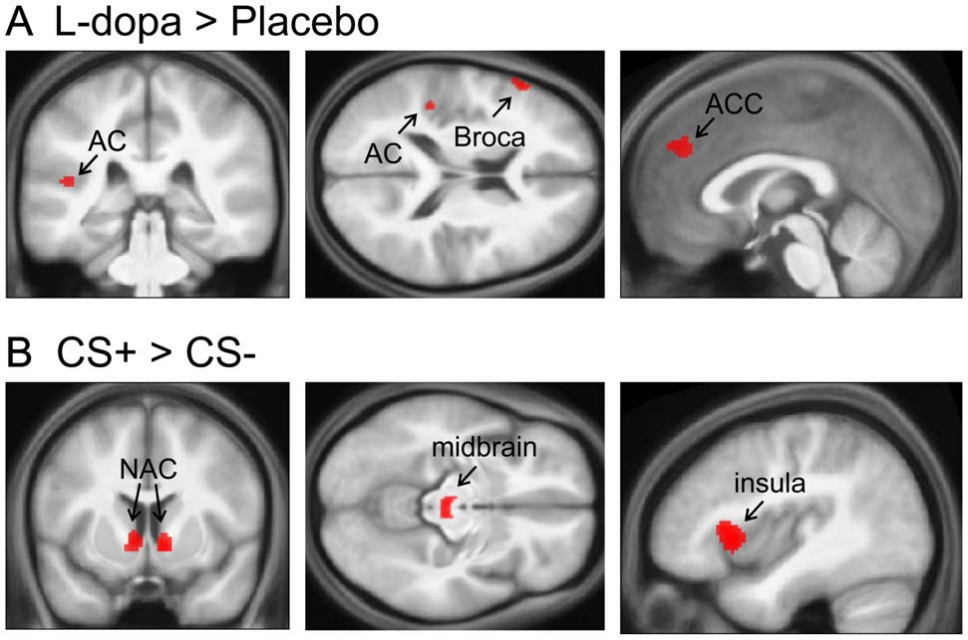
Neural activity for main effect of group (A) and condition (B). Neural activity for (A) main effect of group, which yields stronger activation in the auditory cortex (AC), inferior frontal gyrus (Broca’s area) and anterior cingulate cortex (ACC)/left medial frontal gyrus for the L-dopa treated group compared to the placebo group. (B) Main effect of condition which shows stronger activation for rewarded (CS+) compared to non-rewarded tones (CS−), in several dopaminergic brain areas, such as nucleus accumbens (NAC) and midbrain regions (SN/VTA), as well as insula.

**Figure 4:**
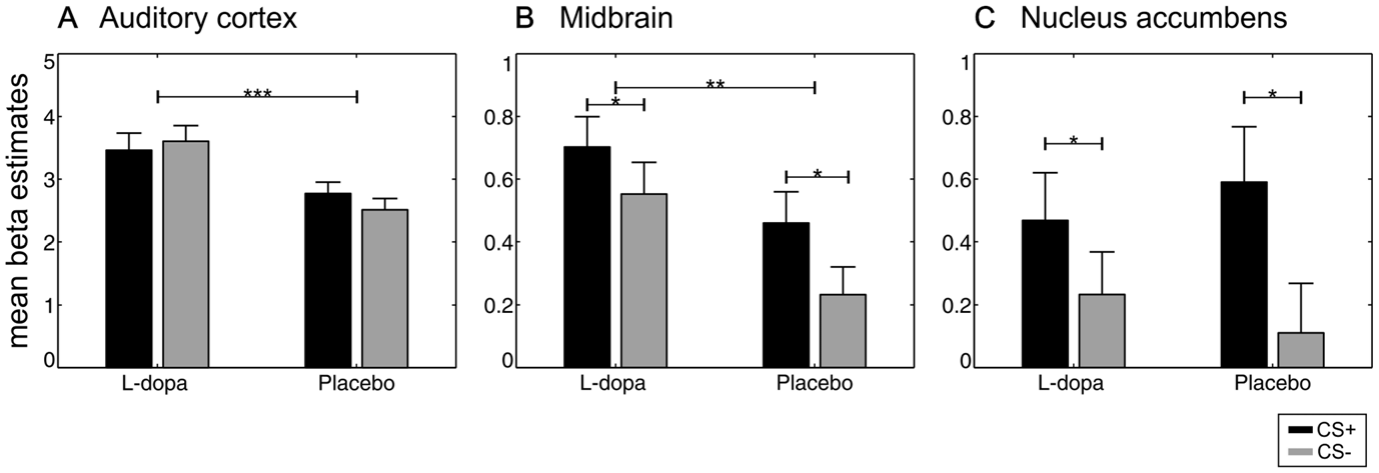
Averaged beta values as a function of group and condition in regions of interest to illustrate the results shown in **Figure 3**. Note that neural activity in the left auditory cortex was significantly enhanced in the L-dopa group compared to the placebo group without any significant differences between CS+ and CS− trials. Differences in CS+ and CS− trials were evident in dopaminergic brain regions (B and C). The plots further illustrates higher neural responses in the midbrain under L-dopa. Black bars show rewarded (CS+) trials, gray bars unrewarded (CS−) trials. Statistically significant differences are marked by asterisks (two factorial ANOVA corrected for multiple comparisons).

We found a significant main effect of group within the left auditory cortex (Figure 3A, 4A, Table 1A), left Broca’s area and anterior cingulate cortex/left superior medial gyrus (Table 2A). Participants receiving L-dopa had significantly increased neural responses in these brain regions. The averaged beta values in Figure 4A illustrate this effect within the left auditory cortex (F(1,106) = 15.87, *p<0.001*). The linear regression analysis provides further evidence that responses to auditory stimuli in left auditory cortex ([x, y, z] = [−54, −38, 22], Z = 3.68, k = 25, see SI Figure S1) are related to the amount of L-dopa plasma levels. Note that the numerically higher neural activity in SN/VTA under L-dopa (F(1,106) = 8.47, *p = 0.004*), evident in the plots in Figure 4B, did not survive corrections for multiple comparisons.

**Table 1:** Results of the full factorial ANOVA with effects of drug treatment (A) and reward anticipation (B).

Contrast	x	y	z	Volume	Z	Region
(A) L-dopa > Placebo	−44	−34	−14	21	3.40	left auditory cortex*
	−58	24	18	75	4.72	left inferior frontal gyrus (Broca’s area)
	0	44	38	184	3.94	ACC/left superior medial gyrus
(B) CS+ > CS−	6	−24	−12	22	3.86	substantia nigra/VTA*
	−8	6	−2	348	4.00	left nucleus accumbens*
	8	12	−4	139	4.25	right nucleus accumbens*
	−40	16	0	375	4.32	left insula
	38	18	8	145	3.96	right insula
	−32	−90	−6	139	4.16	left middle occipital gyrus

**Table 2:** Results of the linear regression analysis using the slope of the individual learning curves: (A) L-dopa group, (B) placebo group and (C) differences between placebo and L-dopa.

Contrast	x	y	z	Volume	Z	Region
(A) L-dopa	−40	−36	12	21	3.46	left auditory cortex*
	52	−12	22	415	4.17	right Rolandic operculum
	−6	−24	40	174	4.08	left middle cingulate cortex
(B) Placebo	−64	−38	20	43	3.89	left auditory cortex*
	−60	−16	4	22	3.41	left auditory cortex*
	58	−10	8	27	3.89	right auditory cortex*
	8	−2	32	1694	4.89	right middle cingulate cortex
	−56	−26	28	524	4.44	left supramarginal gyrus
	32	44	24	456	4.39	right middle frontal gyrus
	−8	−18	−16	748	4.36	VTA/substantia nigra
	52	6	10	234	4.34	right Rolandic operculum
	16	−68	36	111	3.90	right cuneus
	58	−22	28	165	3.86	right supramarginal gyrus
	58	−34	46	72	3.75	right supramarginal gyrus
	−18	−60	32	103	3.73	left precuneus
	32	−14	4	495	3.73	right putamen
(C) Placebo > L-dopa	−8	−18	−14	67	3.97	VTA/substantia nigra
	−16	−88	0	86	3.69	left lingual gyrus

The main effect of condition yielded increased neural activity to CS+ as compared to CS− tones in dopaminergic midbrain (SN/VTA), right and left nucleus accumbens (Figure 3B, 4B–C, Table 1B), bilateral insula and left middle occipital gyrus (Table 1B). Analysis of averaged beta values between rewarded and unrewarded tones in dopaminergic midbrain regions (F(1,106) = 3.84, *p = 0.05*) and nucleus accumbens (F(1,106) = 5.24, *p = 0.02*) illustrates this effect. No group by condition interaction was found, not even at a liberal threshold of *p<0.001*, indicating that effects of L-dopa occurred similarly in CS+ and CS− trials.

**Figure 5:**
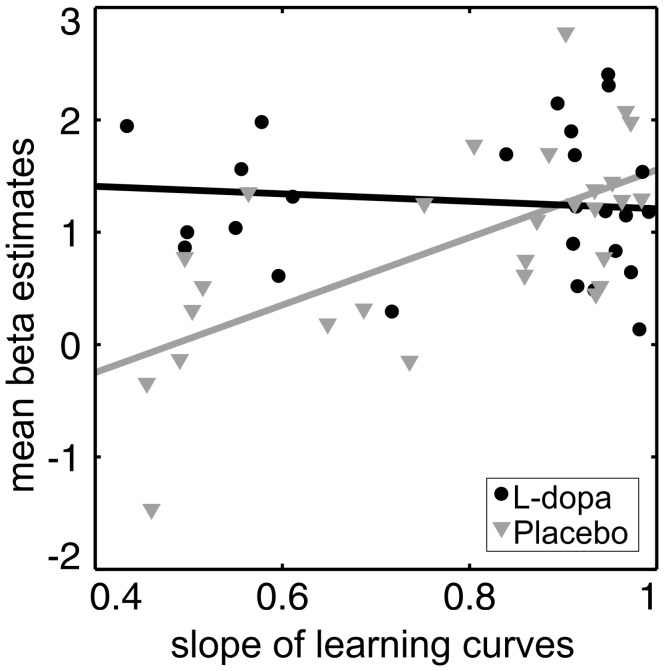
Regression between BOLD activity in dopaminergic midbrain regions and slope of cumulative learning curves. Regression between BOLD activity in dopaminergic midbrain regions (substantia nigra/VTA) and slope of cumulative learning curves: Regression lines show a significant effect for placebo but not for L-dopa.

To investigate whether BOLD activity in subjects with faster learning rates differs from that in subjects with slower learning rates, we performed a linear regression analysis. Table 2 lists brain areas, in which neural responses to FM tones correlated significantly with individual learning rates (i.e. the slope of the learning curves shown in Figure 2A). In both groups, several brain regions showed a higher neural activity in subjects with fast learning rates including, among others, the left auditory cortex, the middle cingulate cortex and in the right Rolandic operculum. Note that the correlation of learning rates and neural activity in the dopaminergic midbrain was only found in the placebo group (F(1,26) = 19.23, *p<0.001*) but absent in subjects treated with L-dopa (F(1,25) = 0.30, *p = 0.5*, see Figure 5). There was no significant effect of learning rates on differential activity to CS+ and CS− in none of the groups.

## Discussion

We aimed to investigate whether dopaminergic stimulation modulates representations of relevant stimuli in human auditory cortex. Our results show that neural activity in the auditory cortex during auditory instrumental learning was similar for reward predicting (CS+) and unrewarded (CS−) tones but generally increased after dopaminergic stimulation. This increase was related to plasma L-dopa levels and learning rate. Behaviorally, dopaminergic stimulation was found to impact on the speed of instrumental responding, especially in unrewarded trials, which were slowed down.

### Neural Effects of Dopaminergic Stimulation

Increasing dopaminergic neurotransmission increased neural activity to FM tones in left auditory cortex, Broca’s area and anterior cingulate cortex. This increase occurred for both reward predicting (CS+) and unrewarded (CS−) FM tones. Note, that lowering the statistical threshold (*p<0.01*, uncorrected) reveals additional activation clusters also in the right auditory cortex for the comparison between the two groups. To test for hemispheric differences we therefore calculated the laterality index (LI-toolbox available in SPM, [59], [60]) for this effect. Results (LI = 0.66±0.01, bootstrapping procedure with 180000 repetitions at *p<0.002* (uncorrected)) suggest a significant left hemispheric predominance for this effect. This lateralization is in line with previous work by Brechmann and Scheich [61] demonstrated a differential task-dependent involvement of the left auditory cortex during categorization of FM tones in humans. They showed an involvement of the left auditory cortex, when participants had to categorize FM tones according to their duration, whereas the right auditory cortex showed higher activation during categorization of FM tone direction. Thus, the dopaminergic modulation of activity in auditory cortex occurred in a region involved in categorizing the specific feature (i.e. duration) of the auditory stimuli. This is also consistent with a learning dependent effect in the left auditory cortex shown previously [28]. The correlation of BOLD activity in left auditory cortex with L-dopa plasma levels further underlines the suggestion that dopamine critically contributes to increases in left auditory cortex activity. A second region shown to be modulated by L-dopa was Broca’s area, which has several functions but has also been linked to frequency discrimination [62] and processing prosodic information [63].

Animal data by Stark and Scheich [20] provides evidence for a release of dopamine in auditory cortex during acquisition of avoidance learning on the first day of training but not on subsequent days. Although there are several differences between avoidance learning paradigms used in animals and the appetitive instrumental learning paradigm used here in humans, we would like to point out that the increases in neural activity in auditory cortex under L-dopa measured in the current study may reflect similar processes as the increase in dopaminergic activity shown in animals with in vivo microdialysis during the first day of avoidance learning. Further studies in animals, which involve learning stages over several days, provide evidence that increased dopaminergic activity at the time point of initial learning promotes later memory formation via gene activation and synaptic remodeling [24], [64]. We were not able to study such long term changes with the current design. However, our data suggest that auditory cortex activity during acquisition is linked to individual learning rates, which suggests that the increased neural activity in auditory cortex under L-dopa may be beneficial for auditory learning. Benefits of dopaminergic stimulation for auditory learning in humans have been shown by Tobey et al. [65], who administered d-amphetamine to patients with cochlear implants and found increased speech tracking scores and neural activity in the auditory cortex. Therefore, our results may be of clinical relevance in situations, where learning of new auditory inputs is required.

Furthermore, Bao et al. [21] showed that the dopaminergic neurotransmitter system is involved in enhancing plasticity in the auditory cortex of rats. They presented a pure tone and simultaneously stimulated the VTA, which resulted in an increased spatial representation of this specific tone in the auditory cortex. Although this study demonstrates that ventral tegmental dopamine-mediated activity enables the reorganization of the auditory cortex, there are several differences between the present study and the study by Bao et al. [21]. These differences include the species (rats vs. humans), the paradigm (classical vs. instrumental conditioning), the auditory stimuli (pure tones vs. FM tones), and the approach taken to stimulate the dopaminergic system (VTA stimulation vs. L-dopa administration). Thus, different results can be due to any of these factors. We suggest however that the lack of differential dopaminergic modulation is primarily due to the different approach of stimulating the dopaminergic system. In the study by Bao and colleages [21] the VTA was stimulated each time a certain tone was presented. Differences between the paired frequency and other frequencies were mapped after the pairing, when no dopaminergic stimulation was present. In contrast, in our experiment, due to the differential conditioning approach and the prior administration of L-dopa, increased dopamine levels were present during presentation of the CS+ and CS−, which may have increased neural activity to both CS+ and CS− tones.

Another region found to be modulated by L-dopa was the anterior cingulate cortex/left superior medial gyrus. This region has been suggested to integrate reward history, evaluate outcomes and select appropriate actions with regard to relevant contextual information [66]–[68] (for a recent review see [69]). The anterior cingulate cortex is modulated by dopaminergic projections from the midbrain, which have been suggested to be responsible for effort related choice behavior [70]. An L-dopa induced modulation of anterior cingulate responses has also been shown in a task investigating cognitive control [71]. In the visual domain it has previously been suggested that reward related activity in the dopamine system impacts on neural activity in anterior cingulate cortex that eventually leads to changes in sensory representation [72]. We suggest that the dopaminergic stimulation induced by L-dopa triggered neural activity in the anterior cingulate cortex, which may have led to changes in auditory cortex activity. Furthermore, Stark et al. [73] measured dopamine release in medial prefrontal cortex with in vivo microdialysis in gerbils. Animals first had to solve an auditory avoidance training in a shuttle box with two different conditioned stimuli in a GO procedure. After some days of training and implantation of a guide cannula for microdialysis in medial prefrontal cortex the relearning experiment started. In the relearning paradigm one of the former GO-condition stimuli was reversed to a NOGO-condition. Within the first session of relearning there was an increased dopamine release in the medial prefrontal cortex in those individuals which rapidly relearned the condition. This study clearly indicates that dopamine release in the medial prefrontal cortex is a key issue for the acquisition of a new behavioral strategy. These results underline the importance of dopamine in the medial prefrontal cortex, where we found stronger activation for the L-dopa treated group compared to the placebo group.

### Neural Effects of Reward Anticipation

Brain activity related to reward anticipation was found in the nucleus accumbens, dopaminergic midbrain regions, and left insula. Activations of dopaminergic brain areas due to reward-predicting stimuli have been shown in several human fMRI studies before [18], [48]–[50], [74]–[76]. According to a study by Daniel and Pollmann [57], nucleus accumbens is especially sensitive to anticipation of a monetary reward.

Given that prior fMRI evidence in humans show increased BOLD activity to reward predicting auditory stimuli [25], [26], [28], we expected to find increased activations in auditory cortex for rewarded, i.e. CS+, as compared to non-rewarded, i.e. CS− tones. In addition, Brosch and colleagues [16] demonstrated that modulation of auditory cortex activity related to reward anticipation can also be observed in the absence of auditory reward predicting stimuli. In their study, monkeys were trained to release a bar when detecting a specific change in auditory stimulation. Repeated correct responses led to an increased reward chance in the next trial. Remarkably, auditory cortex modulations related to the expected reward size were already observed prior to the onset of the auditory stimulation when the monkeys grasp the bar to initiate the next experimental trial. However, our data provides no evidence for such differential activity. A lack of differential activity when using FM tones instead of sine tones may be explained by recent findings showing that task difficulty has a significant effect on auditory cortex plasticity [77]. However, in a previous study by Puschmann et al. [28] using the same FM stimuli, differences in activation between CS+ and CS− tones were found. Comparing the learning curves of the individual subjects of this current study with the previous study by Puschmann et al. [28] indicated that participants here learned much faster than in the previous study. Hence, participants already knew the correct category at an early stage of the experiment and no additional learning occurred. It was shown previously by Stark and Scheich [20] that the dopaminergic system is specifically involved during early phases of associative learning. A study by Reed et al. [78] further suggests that learning related changes in auditory cortex plasticity are not stable. They could show that pairing of tones with stimulation of the cholinergic nucleus basalis induces auditory cortex map plasticity in rats. This plasticity faded over weeks but tone discrimination performance still remained stable. Thus, the data indicate that cortical map expansion improves learning, but is not necessary for good performance after the discrimination task was learned. Even though this study had a different time scale compared to our study, it still demonstrates that plasticity can fade away after learning has taken place. We assume that the differences in auditory cortex activity between the current study and the study by Puschmann et al. [28] may be related to different learning rates and only be observed in early but not later phases of learning.

### Relationship between Neural Activity and Learning

We provide evidence that neural activity in left auditory cortex is related to individual learning curves. The faster a subject learns, the higher is the BOLD activity in this region. This effect was present under L-dopa and placebo. Furthermore, there was a learning related increase in neural activity in the right Rolandic operculum present under L-dopa as well as placebo. This may be explained by the formation of a motor-related circuitry that serves premotor representations for sound production [79].

Within the dopaminergic midbrain only the placebo treated group showed a strong increase in neural activity related to learning whereas neural activity under L-dopa was unrelated to learning (see Figure 5). This result can be explained by the fact that in the drug treated group the dopaminergic system is saturated and is not able to be more activated even with enhanced learning success. De la Fuente-Fernández et al. [80] already showed that 100 mg L-dopa, the same dose as used in this study, is enough to completely saturate the dopaminergic system.

### Behavioral Effects of Dopaminergic Stimulation

There was no difference in RTs after tone presentation for the different conditions and groups which is probably due to the fact that speed was not relevant in this part of the experiment. In contrast, during the number comparison task, where we found RT differences between conditions and groups, participants had to provide fast responses for obtaining a reward.

Dopaminergic stimulation has previously been shown to modulate reward and avoidance learning, word learning and motor plasticity [32], [37], [81], [82]. For example, Ilango et al. [81] suggest that VTA self-stimulation facilitates avoidance learning in animals by an increase of avoidance rate and a decrease in avoidance latency. Pessiglione et al. [32] studied human subjects under dopaminergic stimulation and blockade in an instrumental learning task involving gains and losses and provide evidence that subjects under L-dopa are more likely to choose the most rewarding action. Our paradigm required subjects to categorize FM tones and to subsequently make speedy response to obtain a financial reward, contingent upon trial type. The results provide evidence that dopaminergic stimulation does not impact on learning per se however it does on the speed of instrumental responding depending on trial type. Under L-dopa, decreases in RTs were seen in trials, where a reward could be obtained for fast responding (CS+), whereas increases in RTs were seen in trials in which no reward could be obtained (CS−). Subjects treated with placebo showed increases in RTs in both trial types. In other words dopaminergic stimulation increases the efficiency of responding: Effort is only expended in trials where a reward can be obtained. If no reward can be obtained RTs even increase over the course of the experiment. This finding is in line with suggestions that dopamine is involved in effort related choice behavior [70]. Data by Leyton et al. [83] showed that the ability to respond to stimuli predicting a reward is decreased by decreases in dopamine synthesis and can be prevented by L-dopa. An absence of dopaminergic stimulation on learning per se was also seen in animals Schicknick and colleagues [24] showed that there is no difference between animals receiving D1-like dopamine receptor agonist and vehicle-injected controls during initial training in a FM discrimination task. Only when the animals were retrained hours or days later, there was an enhanced performance in the dopamine treated animals.

### Conclusion

Previous studies in humans already indicated that administration of the dopamine precursor L-dopa facilitates novel word learning, improves motor cortex plasticity in healthy human subjects and motor recovery after stroke [37], [82], [84]. Here, we provide first evidence that dopaminergic stimulation increases neural activity in auditory cortex during instrumental learning.

## Supporting Information

Figure S1 Linear regression using L-dopa blood levels
(PDF)

Table S1 Physiological and subjective measurements
(PDF)
