# Supplementary material for: Effects of L-dopa during Auditory Instrumental Learning in Humans
Source: PLoS One. 2012 Dec 21;7(12):e52504. doi: 10.1371/journal.pone.0052504 (PMC3528678; doi:10.1371/journal.pone.0052504)
Supplement: Figure S1 — Linear regression using L-dopa blood levels (PDF) [file pone.0052504.s001.pdf]

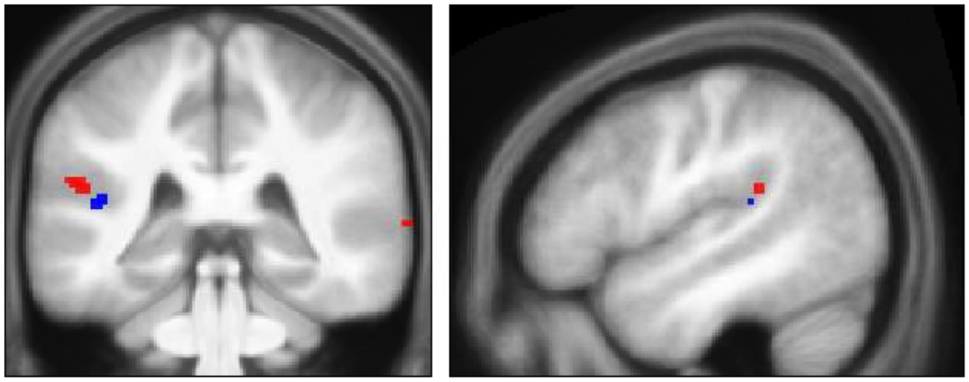

Supporting Figure S1. **Linear regression analysis using the amount of L-dopa blood level as regressor showed significant activation within the left auditory cortex ([x, y, z] = [-54, -38, 22], k = 25, marked in red), which is close to the activation obtained from the main effect of drug treatment in the full factorial ANOVA ([x, y, z] = [-44, -34, 14], k = 21, marked in blue).**
