# Supplementary material for: Effects of L-dopa during Auditory Instrumental Learning in Humans
Source: PLoS One. 2012 Dec 21;7(12):e52504. doi: 10.1371/journal.pone.0052504 (PMC3528678; doi:10.1371/journal.pone.0052504)
Supplement: Table S1 — Physiological and subjective measurements (PDF) [file pone.0052504.s002.pdf]

Factors	Placebo<br>Predrug	Postdrug	L-dopa<br>Predrug	Postdrug
Sedation	27 ± 2.4	22 ± 2.4	24 ± 2.4	22 ± 2.5
Discontendedness	24 ± 2.5	24 ± 2.2	23 ± 2.5	24 ± 2.9
Excitation	28 ± 3.3	27 ± 3.2	21 ± 2.9	25 ± 3.5
Pulse	71.6 ± 1.8	70.7 ± 2.2	69.4 ± 2.0	69.6 ± 1.9
Blood pressure	134.48 ± 2.8 /<br>84.0 ± 2.2	137.70 ± 3.1 /<br>89.1 ± 2.6	135.1 ± 1.7 /<br>86.5 ± 2.0	133.8 ± 2.4 /<br>84.5 ± 1.8

Supporting Table S1: Mood rating scale (Bond and Lader 1974). Subjective ratings (mean and standard error of mean) on visual analogue scales in mm. Pulse and blood pressure before and after intake of drug in both groups.
